# Clinical efficacy of the Galdakao-modified supine Valdivia position vs. the lithotomy position in FANS-UAS-assisted IPC-RIRS for renal calculi

**DOI:** 10.3389/fmed.2026.1886784

**Published:** 2026-09-03

**Authors:** Qiuhua Zhu, Yunxia Cai, An Wang, Jiansheng Xiao, Leming Song, Tairong Liu, Hua Chen

**Affiliations:** 1 Department of Operating Room, Ganzhou People’s Hospital, Jiangxi Medical College, Nanchang University, Ganzhou, Jiangxi, China; 2 Department of Urology, Ganzhou People’s Hospital, Jiangxi Medical College, Nanchang University, Ganzhou, Jiangxi, China; 3 Department of Urology, The Sixth Affiliated Hospital of Jinan University, Dongguan Eastern Central Hospital, Dongguan, China

**Keywords:** Galdakao-modified supine Valdivia position, intelligent pressure-controlled retrograde intrarenal surgery, lithotomy position, renal calculi, retrograde intrarenal surgery, suction ureteral access sheath

## Abstract

**Objective:**

To evaluate the clinical efficacy and safety of a modified Galdakao-modified supine Valdivia (GMSV) position combined with a flexible and navigable suction ureteral access sheath (FANS-UAS) in intelligent pressure-controlled retrograde intrarenal surgery (IPC-RIRS) for the treatment of renal calculi.

**Methods:**

This single-center retrospective cohort study included 158 patients with renal calculi who underwent FANS-UAS-assisted IPC-RIRS between June 2023 and June 2025. Patients were categorized into the GMSV position group (*n* = 78) and conventional lithotomy position group (*n* = 80) according to the intraoperative position used. Operative time, positioning time, stone retrieval basket use, postoperative hospital stay, stone-free rate (SFR) on postoperative day 1 and day 30, reoperation rate, and perioperative complications were compared between groups.

**Results:**

Operative time was significantly shorter in the GMSV group than in the lithotomy group (41.35 ± 7.32 min vs. 55.21 ± 8.25 min, *P* < 0.001). Stone retrieval baskets were required in fewer patients in the GMSV group than in the lithotomy group (3.85% vs. 12.50%, *P* = 0.048). The SFRs on postoperative day 1 were 87.18% and 73.75% in the GMSV and lithotomy groups, respectively, while the corresponding SFRs on postoperative day 30 were 92.31% and 81.25%, respectively. The GMSV group achieved significantly higher SFRs at both time points (both *P* < 0.05). No significant differences were observed between groups in positioning time, postoperative hospital stay, postoperative hemoglobin decrease, reoperation rate, or perioperative complication rate (all *P* > 0.05).

**Conclusion:**

The GMSV position, combined with FANS-UAS-assisted IPC-RIRS, significantly improved the SFR and reduced operative time, while providing similar safety and positioning efficiency compared with the lithotomy position.

## 1 Introduction

Renal stone disease is a prevalent urological condition with a continuously increasing global incidence and substantial healthcare burden. Renal calculi may lead to urinary tract obstruction, recurrent infection, progressive renal impairment, and potentially life-threatening urosepsis, thereby substantially compromising patients’ quality of life (1–3). Current therapeutic approaches for renal calculi include extracorporeal shock wave lithotripsy (ESWL), retrograde intrarenal surgery (RIRS), percutaneous nephrolithotomy (PCNL), and various combined endoscopic techniques (4, 5). Among these modalities, flexible ureteroscopic lithotripsy, the cornerstone technique of RIRS, has been increasingly adopted for the management of a broad spectrum of renal calculi owing to its minimally invasive characteristics, rapid postoperative recovery, and favorable safety profile (6, 7). However, with the expanding application of RIRS in patients with larger stone burdens and anatomically complex collecting systems, optimizing surgical efficiency and achieving satisfactory stone clearance remain significant clinical challenges.

Recent technological advancements in RIRS, including intelligent pressure-controlled systems and negative-pressure suction devices, have been developed to improve operative efficiency, optimize intrarenal conditions, and enhance procedural safety. Intelligent pressure-controlled retrograde intrarenal surgery (IPC-RIRS) enables real-time monitoring and regulation of intrarenal pressure, whereas the flexible and navigable suction ureteral access sheath (FANS-UAS) facilitates continuous evacuation of irrigation fluid and stone fragments, thereby potentially improving surgical visualization and stone-free rate (SFR) (8–10). Nevertheless, previous studies have primarily focused on the efficacy and safety of suction-assisted systems or pressure-control technologies, whereas the impact of patient positioning on the clinical outcomes of FANS-UAS-assisted IPC-RIRS remains largely unexplored. Patient positioning may influence the spatial orientation of the collecting system, gravity-dependent migration of stone fragments, irrigation and suction dynamics, as well as flexible ureteroscope maneuverability during lithotripsy. Compared with the conventional lithotomy position, the Galdakao-modified supine Valdivia (GMSV) position may theoretically facilitate stone fragment evacuation and improve ureteroscope maneuverability during suction-assisted RIRS (11, 12). Therefore, we hypothesized that the GMSV position could enhance stone clearance efficiency and reduce operative duration without increasing the risk of perioperative complications.

The present study aimed to compare the clinical efficacy and safety of the GMSV position and conventional lithotomy position during FANS-UAS-assisted IPC-RIRS for renal calculi. The primary outcomes were operative duration and SFR, whereas secondary outcomes included perioperative complications and other relevant clinical parameters. This study aimed to provide evidence regarding the potential role of patient positioning optimization in improving outcomes during IPC-RIRS.

## 2 Materials and methods

### 2.1 Study population and patient selection

This single-center retrospective cohort study consecutively included patients with renal calculi who underwent IPC-RIRS at our institution between June 2023 and June 2025. Clinical data were collected from the institutional electronic medical record system, operative reports, imaging database, and postoperative follow-up records. The indications for FANS-UAS-assisted IPC-RIRS were determined based on comprehensive clinical assessment, including stone-related symptoms, stone burden, stone location, urinary obstruction, recurrent urinary tract infection, and patient preference. The decision to perform RIRS was made through multidisciplinary clinical evaluation in accordance with current recommendations for the management of renal calculi.

The inclusion criteria were as follows: (1) age ≥18 years; (2) unilateral renal calculi confirmed by preoperative non-contrast computed tomography (CT) with indications for surgical intervention; (3) undergoing FANS-UAS-assisted IPC-RIRS using a 12/14-Fr suction ureteral access sheath; (4) availability of complete preoperative imaging, operative records, and postoperative follow-up data; and (5) hydronephrosis grade ≤2 according to the Grignon grading system. The exclusion criteria were as follows: (1) severe renal insufficiency or uncontrolled urinary tract infection; (2) severe spinal deformity, restricted hip or knee mobility, or other conditions preventing the patient from tolerating the required surgical position; (3) ureteral stricture, severe ureteral tortuosity, or unsuccessful placement of the FANS-UAS during surgery; (4) congenital or acquired renal anatomical abnormalities, including horseshoe kidney, transplanted kidney, or duplicated collecting systems; and (5) incomplete postoperative imaging follow-up within 30 days after surgery.

**Figure 1:**
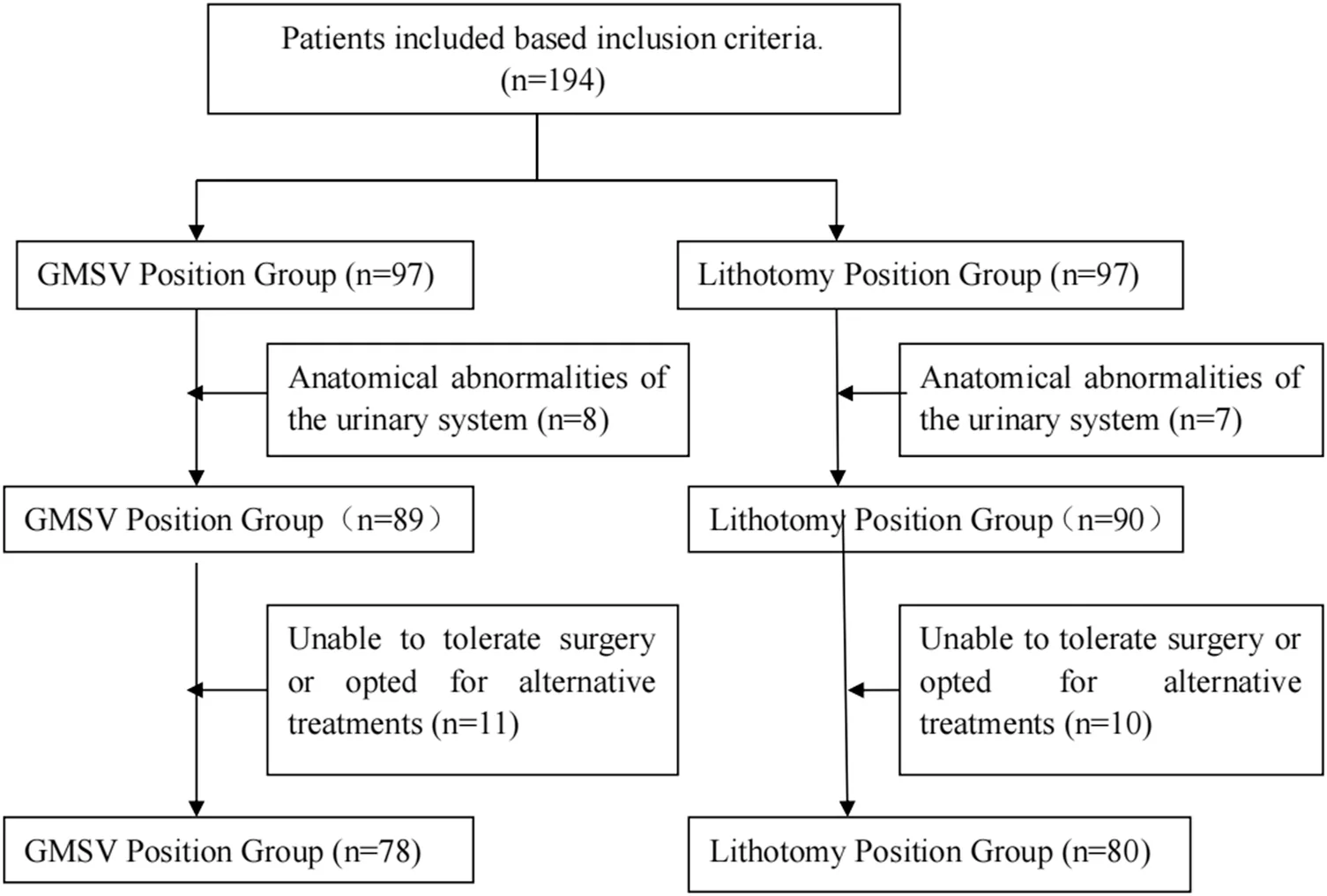
The screening process for this study.

A total of 158 patients were included in the final analysis, including 78 patients in the modified GMSV position group and 80 patients in the conventional lithotomy position group. The patient selection process is illustrated in Figure 1.

### 2.2 Ethical standards

This study was conducted in accordance with the principles of the Declaration of Helsinki and was approved by the Ethics Committee of Ganzhou People's Hospital (Approval No. PJB2024-005-1). Written informed consent for participation in this study was obtained from all patients before data collection. All patient information was anonymized to protect privacy.

### 2.3 Surgical position and perioperative management

**Figure 2:**
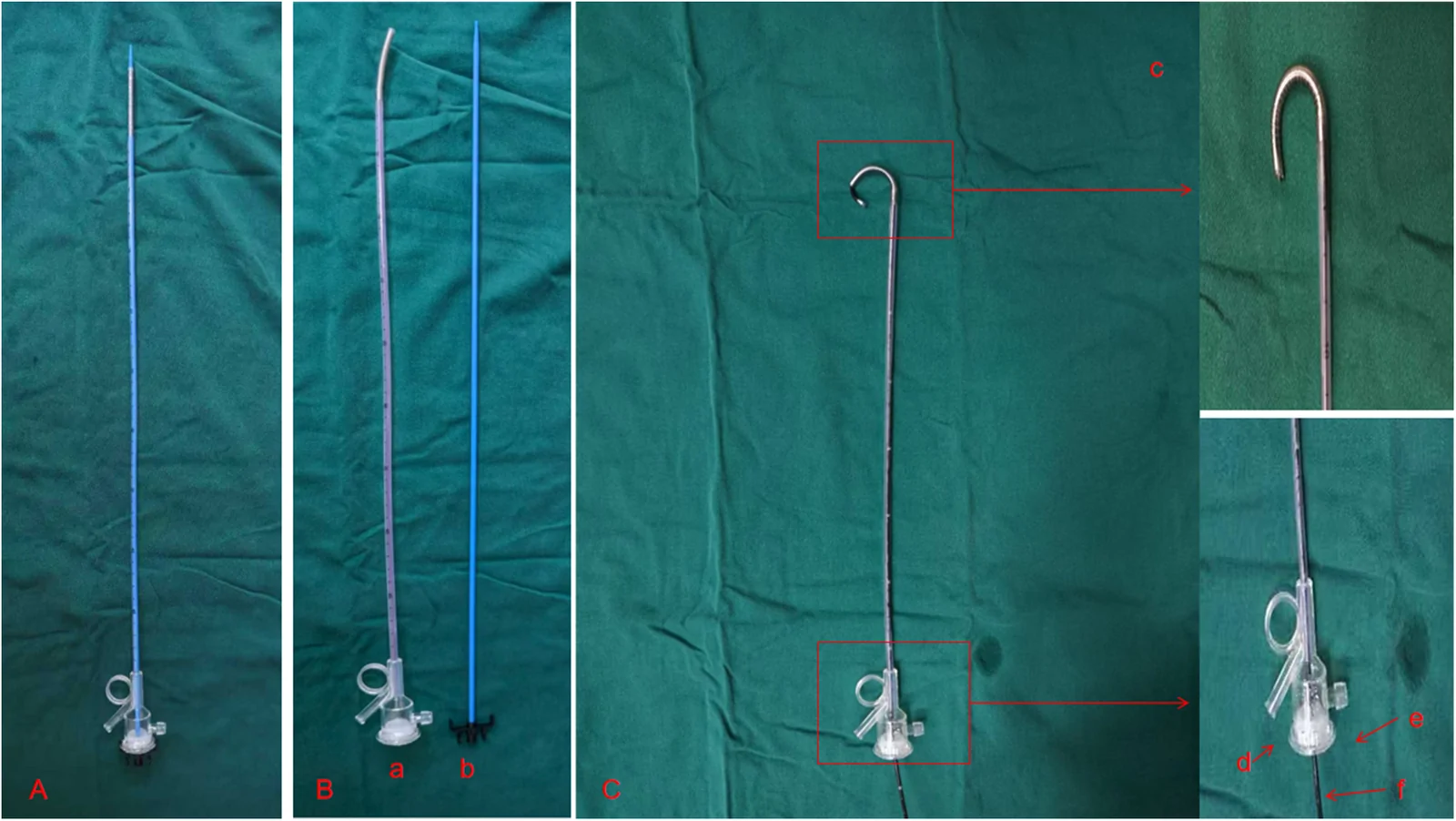
Schematic illustration of the flexible and navigable suction ureteral access sheath (FANS-UAS). **A**: Panoramic view of the FANS-UAS with a deflectable distal segment. **B**: Main body and obturator of FANS-UAS. **C**: Combination of FANS-UAS and a disposable flexible ureteroscope. **a**: Main body FANS-UAS. **b**: Obturator of FANS-UAS. **c**: The distal 5 cm of the FV-UAS is flexible. **d**: Integrated pressure sensing system. **e**: Suction interface. **f**: Disposable digital flexible ureteroscope.

Both positions were routinely used for IPC-RIRS at our center. The choice of position was determined preoperatively by the same surgical team after comprehensive consideration of stone location, stone burden, patient body habitus, hip and knee mobility, anesthetic assessment, and surgeon experience. For the modified GMSV position, a split-leg operating table was used. The patient was placed in a 45°–70° oblique supine position with the affected side upward, and the operating table was adjusted to a 20°–30° Trendelenburg position. The contralateral lower limb was naturally flexed and secured on the leg support. The ipsilateral lower limb was fixed so that the knee joint formed an angle of 80°–90° with the longitudinal axis of the body, with both lower limbs positioned overall at approximately 70° ± 10° in the oblique supine configuration. The sterile instrument table was placed on the left side of the primary surgeon beneath the ipsilateral leg, and the intelligent pressure-control platform was positioned on the dorsal side of the patient (Figures 2A,B). Patients in the conventional lithotomy group were placed in the supine lithotomy position, with both hips flexed at approximately 90° and both lower limbs abducted at approximately 45°. All positioning procedures were performed jointly by uniformly trained operating-room nurses, anesthesiologists, and surgeons. After positioning, pressure-point protection, lower-limb circulation, urinary catheter patency, and the patency of all connecting tubes were checked, and any position-related adverse events were recorded (Figures 2C,D). During the study period, patients underwent IPC-RIRS using either the GMSV position or the conventional lithotomy position according to routine clinical practice. The choice of surgical position was determined based on the operating surgeon's experience, familiarity with the technique, and intraoperative requirements. No randomization was performed because of the retrospective observational design.

### 2.4 Preoperative assessment and perioperative infection management

Stone size, stone density, stone location, stone number, and the degree of hydronephrosis were primarily assessed using preoperative non-contrast computed tomography (NCCT) of the urinary tract. Intravenous urography was used only as an auxiliary examination in selected patients to evaluate urinary tract anatomy, collecting-system morphology, or renal excretory function, and was not used for the primary assessment of stone burden or stone density.

Patients with a negative preoperative urine culture received a single dose of prophylactic antibiotics 30 min before anesthesia induction. Patients with a positive urine culture, pyuria, fever, or elevated inflammatory markers were treated with culture-directed sensitive antibiotics, and surgery was performed only after infection control had been achieved. If pyuria, obviously turbid urine, or inadequate infection control was identified intraoperatively, primary lithotripsy was discontinued. Ureteral stent placement or nephrostomy drainage was performed instead, and second-stage surgery was scheduled after infection control.

**Figure 3:**
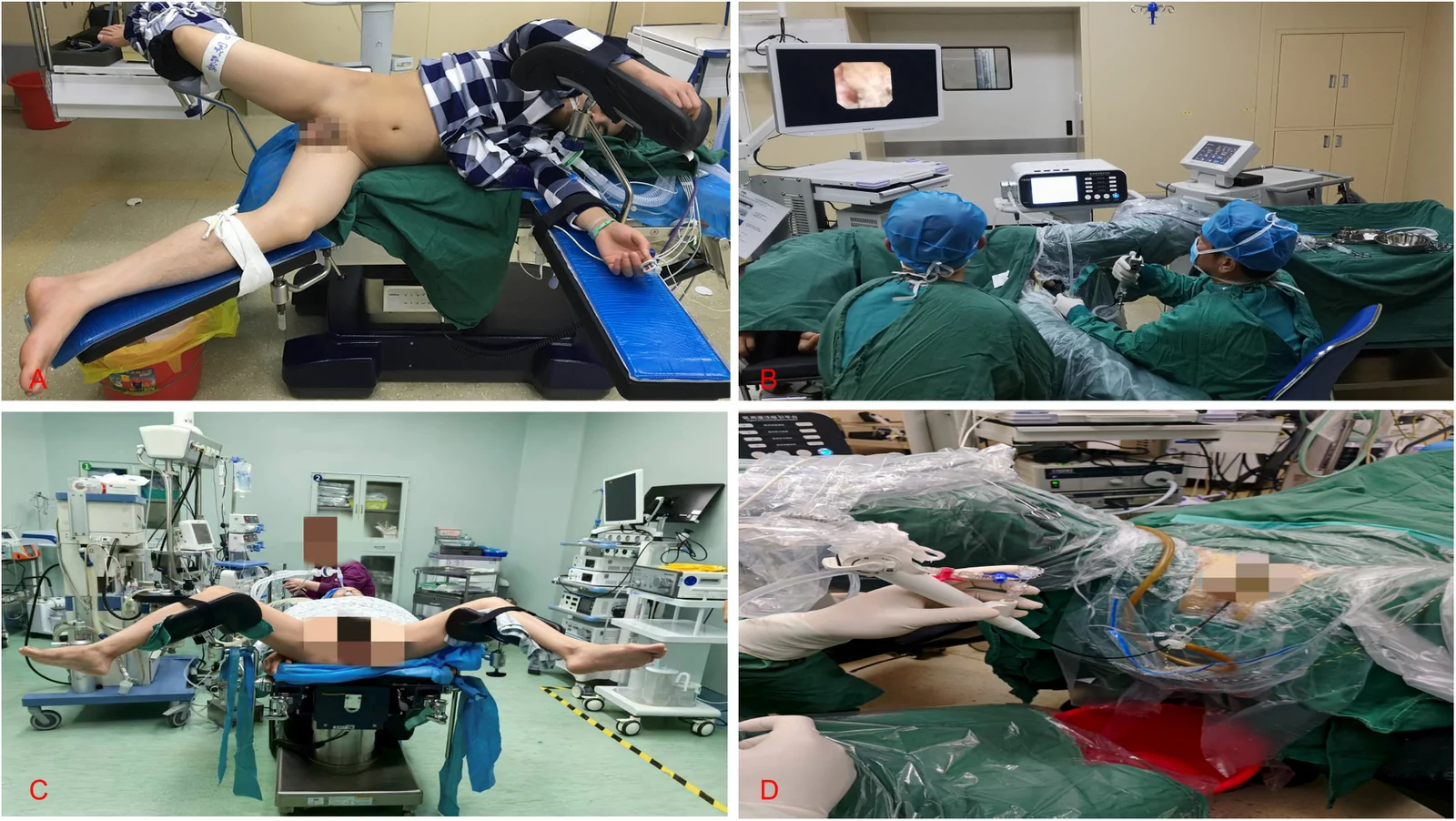
Galdakao-modified supine Valdivia (GMSV) position and lithotomy position, along with corresponding intraoperative images. **A**: Panoramic view of the GMSV position. **B**: Panoramic view of the lithotomy position. **C**: Intraoperative image showing the GMSV position during surgery. **D**: Intraoperative image showing the lithotomy position during surgery.

A 12/14-Fr FANS-UAS (Inventor Technology, Ganzhou, Jiangxi, China) was used intraoperatively (Figure 3). The 12/14-Fr FANS-UAS was selected because it is compatible with an 8.5-Fr digital flexible ureteroscope. This configuration provides sufficient working space for ureteroscopic manipulation while preserving an annular gap between the ureteroscope and the sheath, thereby facilitating continuous negative-pressure aspiration of irrigation fluid, stone dust, and small stone fragments. In addition, this sheath size may reduce compression of the ureteral wall and the risk of sheath-placement-related injury compared with larger-diameter sheaths. An 8.5-Fr digital flexible ureteroscope (11278VU, Karl Storz, Germany) was used in both groups. The intraoperative target intraluminal pressure was set at −5 to −15 mmHg, and the irrigation flow rate was set at 50–150 mL/min. These pressure and irrigation-flow settings were standardized preset parameters for IPC-RIRS at our center and were applied identically in both groups. Therefore, they were described only as technical settings and were not included as variables for between-group statistical comparison.

### 2.5 Surgical procedure

All procedures were performed under general anesthesia by the same experienced endourologist. Before the study period, the surgical team underwent standardized training in IPC-RIRS procedures, FANS-UAS usage, operation of the intelligent irrigation–suction pressure-control platform, and patient positioning techniques. After completion of patient positioning, a semirigid ureteroscope was initially advanced into the ureter and renal pelvis. After confirming ureteral patency and excluding significant ureteral stricture, a Zebra guidewire was inserted. A 12/14-Fr FANS-UAS (Inventor Technology, Ganzhou, Jiangxi, China) was subsequently advanced over the guidewire. The pressure-monitoring and suction ports of the sheath were connected to the intelligent irrigation–suction pressure-control platform via the corresponding pressure-monitoring and suction tubes, respectively. The platform was activated after completion of zero calibration. After introduction of the flexible ureteroscope through the sheath, the renal pelvis and all calyces were systematically inspected for stone localization. Lithotripsy was performed using a holmium:YAG laser with a 200-*μ*m laser fiber. The laser settings were standardized at a pulse energy of 0.8 J, frequency of 25 Hz, and output power of 20 W, with dusting used as the primary lithotripsy strategy. During lithotripsy, fine stone dust and small fragments were continuously evacuated through the space between the ureteroscope and access sheath under negative-pressure suction. Relatively larger fragments were aspirated during gradual withdrawal of the ureteroscope. A stone retrieval basket was not routinely used and was reserved for cases in which larger fragments could not be effectively aspirated through the sheath, impacted calyceal stones required mobilization, or lower-pole stones required relocation.

At the completion of the procedure, a double-J ureteral stent and urinary catheter were routinely placed. The timing of double-J stent removal was determined based on postoperative recovery, residual stone status, and infection risk, and was generally scheduled 2–4 weeks after surgery. The surgical technique, laser settings, irrigation parameters, and postoperative management protocols were identical between the two groups.

### 2.6 Outcome measures and definitions

Operative time was defined as the interval from insertion of the semirigid ureteroscope into the urethra to completion of double-J stent placement and removal of the endoscope. Positioning time was defined as the interval from initiation of patient positioning after successful induction of anesthesia to completion of position fixation and preparation before surgical disinfection and draping.

The primary outcomes were the SFR on postoperative day 1 and postoperative day 30. Secondary outcomes included operative time, positioning time, intraoperative blood loss, postoperative hospital stay, perioperative complications, reoperation rate, and stone composition analysis. Because of the retrospective design, continuous intrarenal pressure curves, total irrigation volume, laser activation time, and cumulative laser energy were unavailable for all patients and were therefore not included in the final analysis. Residual stones were assessed using NCCT performed on postoperative days 1 and 30. Preoperative and postoperative images were independently reviewed by two urologists or radiologists. In cases of disagreement, a third senior physician reviewed the images and made the final decision. Residual fragments ≤2 mm without associated obstruction, infection, or symptoms were defined as clinically insignificant residual fragments (8).

SFR was categorized according to residual fragment size as follows: grade A, no residual stones; grade B, residual fragments measuring 0–2 mm; grade C, residual fragments measuring 2.1–4 mm; and grade D, residual fragments >4 mm (8). Grades A and B were considered to meet the clinical stone-free criterion, whereas grades C and D were classified as residual stone status. Perioperative complications were assessed according to the Clavien–Dindo classification system (13). The occurrence of intraoperative complications, postoperative fever, urosepsis, ureteral injury, hematuria, pain, and position-related nerve or joint injuries was recorded. Postoperative fever was defined as a body temperature ≥38.0 °C. Urosepsis was diagnosed based on infection-related clinical manifestations, laboratory findings, and clinical judgment.

### 2.7 Statistical analysis

Statistical analyses were performed using IBM SPSS Statistics version 26.0 (IBM Corp., Armonk, NY, USA). Continuous variables were assessed for normality using the Shapiro–Wilk test. Normally distributed variables are presented as mean ± standard deviation (SD) and were compared between groups using the independent-samples t-test. Non-normally distributed continuous variables are presented as median (interquartile range, IQR) and were analyzed using the Mann–Whitney U test. Categorical variables are expressed as number (percentage) and were compared using the chi-square test or Fisher's exact test, as appropriate. Missing data were assessed before statistical analysis. Because this was a retrospective study based on complete medical records, variables with missing information were excluded from the corresponding analyses, and no data imputation was performed. For multiple comparisons involving related outcomes, the statistical interpretation was based on predefined primary and secondary endpoints, and no additional adjustment was applied. A two-sided *P* value < 0.05 was considered statistically significant.

## 3 Results

### 3.1 Demographic and baseline characteristics

A total of 158 patients were included in the final analysis, comprising 78 patients in the GMSV group and 80 patients in the lithotomy group. Baseline demographic and clinical characteristics were well balanced between the two groups, with no statistically significant differences observed in demographic characteristics, comorbidities, or stone-related parameters (all *P* > 0.05, Table 1).

**Table 1:** Demographic and baseline characteristics of patients in the GMSV and lithotomy groups.

Characteristics	GMSV Position Group	Lithotomy Position Group	t/*χ*2	*P* value
Number of patients, (n)	78	80		
Age (years), mean ± SD	48.62 ± 13.23	49.36 ± 12.58	0.360	0.719
Gender, *n* (%)			0.110	0.740
Male	43 (55.13)	42 (52.50)	0.017	0.896
Female	35 (44.87)	38 (47.50)		
BMI^a^ (kg/m^2^), mean ± SD	23.21 ± 4.17	23.25 ± 4.35	0.059	0.953
Comorbidities				
Hypertension	10 (12.82)	11 (13.75)	0.030	0.863
Diabetes mellitus	7 (8.97)	8 (10.00)	0.048	0.830
Preoperative hemoglobin (g/L)	132.5 ± 20.9	133.6 ± 19.5	0.342	0.733
Stone characteristics				
Stone size (mm), mean ± SD	15.21 ± 4.32	15.36 ± 4.65	0.210	0.834
Stone density (Hu), mean ± SD	802.6 ± 262.1	798.8 ± 265.3	0.091	0.928
Operated side, *n* (%)			0.107	0.744
Left	36 (46.15)	39 (48.75)		
Right	42 (53.85)	41 (51.25)		
Previous ipsilateral stone surgery, *n* (%)	8 (10.26)	9 (11.25)	0.041	0.840
RIRS	5 (6.41)	6 (7.50)		
PCNL	2 (2.56)	2 (2.50)		
Open surgery	1 (1.28)	1 (1.25)		
Stone location, *n* (%)			0.210	0.976
Renal pelvis	18 (23.08)	17 (21.25)		
Upper calyx	0 (0.00)	0 (0.00)		
Middle calyx	12 (15.38)	13 (16.25)		
Lower calyx	10 (12.82)	9 (11.25)		
Multiple calyces	38 (48.72)	41 (51.25)		
Hydronephrosis grade, *n* (%)			0.409	0.815
No	35 (44.87)	38 (47.50)		
Mild	21 (26.92)	23 (28.75)		
Moderate	22 (28.21)	19 (23.75)		
Severe	0 (0.00)	0 (0.00)		
Urine culture, *n* (%)			0.022	0.882
Positive	12 (15.38)	13 (16.25)		
Negative	66(84.62)	67(83.75)		

a BMI, body mass index; RIRS, retrograde intrarenal surgery; PCNL, percutaneous nephrolithotomy.

### 3.2 Intraoperative and postoperative outcomes

Operative time was significantly shorter in the GMSV group than in the lithotomy group (41.35 ± 7.32 min vs. 55.21 ± 8.25 min, *P* < 0.001). The proportion of patients requiring stone retrieval baskets was significantly lower in the GMSV group than in the lithotomy group (3.85% vs. 12.50%, *P* = 0.048). No significant differences were observed in postoperative hospital stay, secondary procedures, or postoperative hemoglobin levels between the two groups (all *P* > 0.05; Table 2). Regarding the primary outcomes, the SFR was significantly higher in the GMSV group on postoperative day 1 (87.18% vs. 73.75%, *P* = 0.034) and remained significantly higher at 1 month after surgery (92.31% vs. 81.25%, *P* = 0.041). The overall perioperative complication rates were 10.26% and 15.00% in the GMSV and lithotomy groups, respectively, with no significant difference between groups (*P* = 0.574). No significant differences were observed in position-related complications or individual postoperative complications between the groups (Table 2).

**Table 2:** Intraoperative and postoperative outcomes between the GMSV and lithotomy groups.

Characteristics	GMSV Position Group	Lithotomy Position Group	t/χ2	*P* value
Number of patients, (*n*)	78	80		
Operative time (min)	41.35 ± 7.32	55.21 ± 8.25	11.160	＜0.001
Use of stone retrieval baskets, *n* (%)	3 (3.85)	10 (12.50)	3.917	0.048
Postoperative hospital stay (days), mean ± SD	2.12 ± 0.65	2.31 ± 0.62	1.880	0.062
SFR on postoperative day 1 (Grades A + B), *n* (%)	68 (87.18)	59 (73.75)	4.516	0.034
SFR at 1 month after surgery (Grades A + B), *n* (%)	72 (92.31)	65 (81.25)	4.190	0.041
Secondary procedures, *n* (%)	1 (1.28)	2 (2.50)	0.001	0.982
Postoperative hemoglobin level, (g/L)	130.2 ± 19.3	131.8 ± 19.9	0.513	0.609
Total position-related complications	2 (2.56)	3 (3.75)	0.001	0.977
Skin injury	1 (1.28)	1 (1.25)		
Joint injury	1 (1.28)	0 (0.00)		
Nerve injury	0 (0.00)	2 (2.50)		
Overall complications^a^, *n* (%)	8 (10.26)	12 (15.00)	0.316	0.574
Grade I				
Fever (≥38.0 °C)	2 (2.56)	3 (3.75)	0.001	0.977
Nausea/vomiting	4 (5.13)	6 (7.50)	0.082	0.775
Subcapsular renal hematoma	1 (1.28)	2 (2.50)	0.001	0.974
Persistent hematuria	2 (2.56)	3 (3.75)	0.001	0.977
Grade II				
Pleural effusion	0 (0.00)	0 (0.00)	-	-
Ureteral contusion	1 (1.28)	2 (2.50)	0.001	0.974
Blood transfusion	0 (0.00)	0 (0.00)	-	-
Grade III-IV				
Steinstrasse (Grade III)	0 (0.00)	0 (0.00)	-	-
Ureteral perforation (Grade IV)	0 (0.00)	0 (0.00)	-	-
Sepsis (Grade IV)	1 (1.28)	2 (2.50)	0.001	0.974
Stone composition				
Calcium oxalate stones, *n* (%)	59（75.64）	60（75.00）	0.009	0.926
Infection-related stones, *n* (%)	10（12.82）	11（13.75）	0.030	0.863
Uric acid stones, *n* (%)	7（8.97）	7（8.75）	0.003	0.960
Cystine stones, *n* (%)	2（2.56）	2（2.50）	0.231	0.631

VAS, visual analogue scale.

Based on the Clavien–Dindo classification system, some patients experienced multiple complications simultaneously.

a SFR was graded as follows: Grade A (no residual fragments), Grade B (residual fragments 0–2 mm), Grade C (residual fragments 2.1–4 mm), and Grade D (residual fragments > 4 mm).

## 4 Discussion

Despite continuous advances in flexible ureteroscopes, ureteral access sheaths, and suction-assisted technologies, achieving efficient stone clearance remains challenging in patients undergoing RIRS for complex renal calculi. Prolonged operative time, stone fragment migration, and residual fragments remain important factors affecting surgical outcomes (14–16). Therefore, in addition to technological innovations, optimization of procedural conditions, including patient positioning, may represent a potential strategy to further improve surgical efficiency and stone clearance (16–18). In the present study, we compared the clinical outcomes of the modified GMSV position and the conventional lithotomy position during FANS-UAS-assisted IPC-RIRS. Our findings demonstrated that patients treated in the modified GMSV position experienced shorter operative times and higher SFRs on postoperative day 1 and day 30, without an increase in perioperative complications. These results suggest that, under standardized IPC-RIRS and FANS-UAS conditions, patient positioning may represent an additional factor influencing operative efficiency and SFR.

The theoretical basis of the GMSV position originates from its application in supine PCNL and ECIRS. Valdivia-Uría et al. first demonstrated the feasibility of supine PCNL and highlighted its advantages in avoiding patient repositioning and facilitating anesthetic management (19). Subsequently, Ibarluzea et al. modified this position to allow simultaneous antegrade and retrograde endoscopic access (20). Scoffone et al. further reported the application of GMSV position in ECIRS and emphasized its benefits in surgical exposure and endoscopic maneuverability during complex stone treatment (21). Although previous studies mainly focused on PCNL or ECIRS, these findings regarding renal spatial orientation and gravity-assisted drainage provide a theoretical rationale for applying the GMSV position in suction-assisted RIRS.

The potential benefits of the GMSV position may be related to its influence on the spatial relationship among the collecting system, ureteral access sheath, and stone fragments (22, 23). In the conventional lithotomy position, stone fragments may remain distributed within different calyces, particularly the lower pole, where clearance can be limited by unfavorable infundibular anatomy and restricted ureteroscopic access angles (24). Through trunk inclination and modified lower-limb positioning, the GMSV position may alter the relative orientation of the renal collecting system and facilitate the movement of fragments toward the renal pelvis and ureteral access sheath. Under the combined effects of gravity, irrigation flow, and negative-pressure suction, this spatial configuration may promote fragment evacuation and reduce repeated endoscopic manipulation.

The effectiveness of FANS-UAS-assisted clearance depends not only on the suction capability of the device but also on fragment distribution, irrigation dynamics, collecting system anatomy, and the spatial relationship between the sheath tip and residual fragments (25, 26). In this context, the modified GMSV position may enhance the utilization of negative-pressure suction by optimizing fragment accessibility. The shorter operative time and improved SFR observed in the GMSV group may therefore be associated with improved ureteroscopic maneuverability, reduced repeated repositioning of fragments, and more efficient evacuation through the suction sheath. However, these proposed mechanisms remain theoretical, and further studies incorporating intraoperative imaging assessment or quantitative evaluation of collecting system orientation are required to validate these assumptions.

Regarding perioperative safety, the GMSV position maintains characteristics of the supine or oblique supine position and may provide advantages in airway accessibility, respiratory and circulatory monitoring, and anesthetic management compared with prone positioning (27). Although the GMSV position requires more complex positioning procedures than conventional lithotomy, no increase in overall perioperative complications or position-related complications was observed in the present study. These findings indicate that, when appropriate positioning techniques, fixation methods, and pressure-point protection are applied, the modified GMSV position appears to be a feasible and safe option for IPC-RIRS.

From the perspective of surgical ergonomics, the may provide a more favorable working environment by creating relatively independent operating spaces for the surgeon, assistant, instruments, and irrigation–suction system (27, 28). This configuration may facilitate intraoperative monitoring of pressure changes, adjustment of irrigation and suction parameters, and flexible ureteroscope manipulation. Nevertheless, careful attention should be paid to lower-limb positioning, hip and knee joint protection, and prevention of prolonged nerve compression to minimize potential position-related complications.

It should be emphasized that the findings of this study should be interpreted cautiously. Although the GMSV position was associated with improved operative efficiency and stone clearance, no significant difference in perioperative complication rates was observed between the two groups. Therefore, our findings do not indicate that the GMSV position universally reduces complications, but rather suggest that it may provide additional technical advantages for selected patients undergoing FANS-UAS-assisted IPC-RIRS.

A major strength of this study is that all patients underwent the same FANS-UAS-assisted IPC-RIRS protocol, minimizing the influence of different surgical devices and operative techniques on the comparison of patient positioning. Nevertheless, several limitations should be acknowledged. First, this was a single-center retrospective cohort study with a relatively limited sample size, and residual confounding factors could not be completely excluded despite comparable baseline characteristics between groups. Second, patient positioning was determined according to clinical practice and surgeon preference rather than random allocation, introducing potential selection bias. Third, multivariable adjustment was not performed, and therefore the observed association between patient positioning and clinical outcomes cannot establish a causal relationship. Fourth, several intraoperative parameters, including continuous intrarenal pressure curves, total irrigation volume, laser activation time, and cumulative laser energy, were unavailable because of the retrospective design. Future prospective multicenter studies with larger sample sizes and comprehensive adjustment for potential confounders are warranted to further clarify the independent contribution of patient positioning to outcomes following IPC-RIRS.

## 5 Conclusion

In patients undergoing FANS-UAS-assisted IPC-RIRS for renal calculi, the modified GMSV position was associated with shorter operative time and higher short-term SFR compared with the conventional lithotomy position, without increasing perioperative or position-related complications. The potential benefits of the modified GMSV position may be related to improved spatial coordination between the collecting system, ureteral access sheath, and stone fragments, thereby facilitating fragment evacuation under gravity-assisted movement and negative-pressure suction. Further prospective studies are required to validate these findings and determine the independent role of patient positioning in optimizing RIRS outcomes.

## Data Availability

The datasets presented in this study can be found in online repositories. The names of the repository/repositories and accession number(s) can be found in the article/Supplementary Material.

## References

[1] Sorokin I Mamoulakis C Miyazawa K Rodgers A Talati J Lotan Y. Epidemiology of stone disease across the world. World J Urol. (2017) 35(9):1301–20. 10.1007/s00345-017-2008-6 28213860

[2] Bargagli M Scoglio M Howles SA Fuster DG. Kidney stone disease: risk factors, pathophysiology and management. Nat Rev Nephrol. (2025) 21(11):794–808. 10.1038/s41581-025-00990-x 40790363

[3] Prochaska ML Zisman AL. Nephrolithiasis. Adv Kidney Dis Health. (2024) 31(6):529–37. 10.1053/j.akdh.2024.08.007 39577887 PMC13561066

[4] Setthawong V Srisubat A Potisat S Lojanapiwat B Pattanittum P. Extracorporeal shock wave lithotripsy (ESWL) versus percutaneous nephrolithotomy (PCNL) or retrograde intrarenal surgery (RIRS) for kidney stones. Cochrane Database Syst Rev. (2023) 8(8):Cd007044. 10.1002/14651858.CD007044.pub4 37526261 PMC10392035

[5] Chen Y Cui X Peng Z Wang S Zhou H Qi Y. Efficacy and safety of tip-bendable suction ureteral access sheath-assisted flexible ureteroscopic lithotripsy compared with mini-percutaneous nephrolithotomy for the management of 1–3 cm simple lower calyceal calculi: a prospective randomized controlled trial. World J Urol. (2025) 43(1):410. 10.1007/s00345-025-05792-1 40616669

[6] Xie Y Gong H Zheng Q Ding M Shi Z Guan B. Efficacy and safety of flexible and navigable suction access sheaths versus traditional access sheath in flexible ureteroscopic lithotripsy: a systematic review and meta-analysis. World J Urol. (2025) 43(1):487. 10.1007/s00345-025-05856-2 40796988

[7] Multescu R Geavlete P Georgescu D Surcel C Bulai C Mares C. Temperature increase during flexible ureteroscopic approach with holmium Laser lithotripsy: how much should we be concerned? Medicina (Kaunas, Lithuania). (2025) 61(8):1335. 10.3390/medicina61081335 40870380 PMC12387338

[8] Gauhar V Traxer O Castellani D Sietz C Chew BH Fong KY. Could use of a flexible and navigable suction ureteral access sheath be a potential game-changer in retrograde intrarenal surgery? Outcomes at 30 days from a large, prospective, multicenter, real-world study by the European association of urology urolithiasis section. Eur Urol Focus. (2024) 10(6):975–82. 10.1016/j.euf.2024.05.010 38789313

[9] Xiao J Chen H Ge J Kuang J Huang X Zhai Q. Clinical efficacy analysis of two different types of ureteral access sheaths in RIRS for the treatment of 2–4 cm renal stones. World J Urol. (2025) 43(1):381. 10.1007/s00345-025-05776-1 40542192

[10] HUANG X SONG L DENG X CHEN H XIAO J KUANG J. Comparative efficacy and safety of intelligent pressure-controlled versus flexible vacuum-assisted ureteral access sheath for 2–4 cm renal calculi. Minerva Urol Nephrol. (2026) 78(1):136–44. 10.23736/S2724-6051.24.05814-2 39093224

[11] Otsuka I Terada N Iwamoto H Kobayashi T Kamoto T. Comparison of safety and efficacy in endoscopic combined intrarenal surgery performed in the lateral decubitus and galdakao-modified supine Valdivia positions. Urology. (2023) 172:49–54. 10.1016/j.urology.2022.10.018 36370770

[12] Abouelgreed TA Abdelaal MA Amin MM Elatreisy A Shalkamy O Abdrabuh AM. Endoscopic combined intrarenal surgery in the prone split-leg position versus galdakao-modified supine Valdivia position for the management of partial staghorn calculi. BMC Urol. (2022) 22(1):163. 10.1186/s12894-022-01115-3 36266713 PMC9583483

[13] Clavien PA Barkun J De Oliveira ML Vauthey JN Dindo D Schulick RD. The clavien-dindo classification of surgical complications: five-year experience. Ann Surg. (2009) 250(2):187–96. 10.1097/SLA.0b013e3181b13ca2 19638912

[14] Duan H Xu B Xie T. Flexible negative pressure suction ureteral access sheath combined with ureteroscopy in the treatment of upper urinary tract calculi: a meta-analysis. Medicine (Baltimore). (2025) 104(47):e45933. 10.1097/MD.0000000000045933 41305827 PMC12643778

[15] Wang F Tang Y Xiao B Abudulikemu S Li J. Comparison of tip-flexible negative pressure suction sheath and conventional sheath in pediatric renal calculi RIRS: the Hotan experience. Urolithiasis. (2025) 53(1):193. 10.1007/s00240-025-01875-1 41081901

[16] Papatsoris A Geavlete B Radavoi GD Alameedee M Almusafer M Ather MH. Management of urinary stones by experts in stone disease (ESD 2025). Arch Ital Urol Androl. (2025) 97(2):14085. 10.4081/aiua.2025.14085 40583613

[17] Zhang J Dai L Xu J Song R Meng X Shi H. To investigate the effects of surgical position on intrarenal pressure and intrarenal temperature during retrograde intrarenal flexible ureteroscopic lithotripsy: an *in vitro* model. Urolithiasis. (2025) 53(1):145. 10.1007/s00240-025-01817-x 40719769 PMC12304029

[18] Yang Z Zhai Q Wu J Song L Huang Y Sun T. Intelligent pressure-controlled retrograde intrarenal surgery versus microchannel percutaneous nephrolithotomy to treat 2–3 cm renal calculi. Urolithiasis. (2025) 53(1):129. 10.1007/s00240-025-01799-w 40580337 PMC12206191

[19] Uria J Gerhold J Lopez J Rodriguez S Navarro C Fabian M. Technique and complications of percutaneous nephroscopy: experience with 557 patients in the supine position. J Urol. (1998) 160(6 Pt 1):1975–8. 10.1016/S0022-5347(01)62217-1 9817303

[20] Ibarluzea G Scoffone CM Cracco CM Poggio M Porpiglia F Terrone C. Supine Valdivia and modified lithotomy position for simultaneous anterograde and retrograde endourological access. BJU Int. (2007) 100(1):233–6. 10.1111/j.1464-410X.2007.06960.x 17552975

[21] Scoffone CM Cracco CM Cossu M Grande S Poggio M Scarpa RM. Endoscopic combined intrarenal surgery in galdakao-modified supine Valdivia position: a new standard for percutaneous nephrolithotomy? Eur Urol. (2008) 54(6):1393–403. 10.1016/j.eururo.2008.07.073 18715696

[22] Xu Z- Du G- Zhao Y- Wang H- Chen G- Tao C. Single-center experience of micro-perc in the treatment of children with 1.0–2.0 cm sized kidney stones in the galdakao-modified supine Valdivia position. World J Urol. (2023) 41(3):837–41. 10.1007/s00345-023-04289-z 36749393

[23] Ozgur G Gokmen E Tanidir Y Cam K Sener TE. Surgical success and predictive factors for residual stones following supine percutaneous nephrolithotomy in the galdakao-modified Valdivia position. Medeni Med J. (2025) 40(4):217–25. 10.4274/MMJ.galenos.2025.59265 41479155 PMC12758505

[24] Çanakcı C Dinçer E Can U Coşkun A Otbasan BK Özkaptan O. The relationship between stone-free and patient position in retrograde intrarenal surgery: a randomized prospective study. World J Urol. (2024) 42(1):308. 10.1007/s00345-024-05013-1 38722376 PMC11081972

[25] Hamamoto S Okada S Inoue T Taguchi K Kawase K Okada T. Comparison of the safety and efficacy between the prone split-leg and galdakao-modified supine Valdivia positions during endoscopic combined intrarenal surgery: a multi-institutional analysis. Int J Urol. (2021) 28(11):1129–35. 10.1111/iju.14655 34342062

[26] Wang D Sun H Xie D Liu Z Yu D Ding D. Application of a new position in endoscopic combined intrarenal surgery: modified prone split-leg position. BMC Urol. (2022) 22(1):38. 10.1186/s12894-022-00994-w 35305626 PMC8934476

[27] Cracco CM Scoffone CM Poggio M Scarpa RM. The patient position for PNL: does it matter? Arch Ital Urol Androl. (2010) 82(1):30–1. 20593714

[28] Testa V Père M Alliot H Defert C Braik K Amar S. Comparison of the outcomes of mini-percutaneous nephrolithotomy for kidney stones in galdakao modified position between preschoolers and schoolchildren: a single-centre study. J Pediatr Surg. (2026) 61(3):162913. 10.1016/j.jpedsurg.2025.162913 41500312

